# Comparative Chemical Space Analysis of Pesticides and Substances with Genotoxicity Data

**DOI:** 10.1021/acs.chemrestox.5c00198

**Published:** 2025-10-29

**Authors:** Daniel H. Foil, Jeannette König, Kristin Herrmann, Roxane Axel Jacob, Carsten Kneuer, Johannes Kirchmair

**Affiliations:** † Department of Pesticides Safety, German Federal Institute for Risk Assessment, Max-Dohrn-Str. 8-10, 10589 Berlin, Germany; ‡ Vienna Doctoral School of Pharmaceutical, Nutritional and Sport Sciences, 27258 University of Vienna, Josef-Holaubek-Platz 2, 1090 Vienna, Austria; § Department of Pharmaceutical Sciences, Division of Pharmaceutical Chemistry, Faculty of Life Sciences, 27258 University of Vienna, Josef-Holaubek-Platz 2, 1090 Vienna, Austria

## Abstract

Experimental genotoxicity data are required for pesticidal and biocidal active substances prior to regulatory approval, while for their metabolites and impurities, in silico predictions are often accepted. Nonetheless, the extent to which these compounds are represented in publicly available genotoxicity databases remains unclear. Herein, we utilize chemical space methods to define the overlap between pesticide substances (active substances, metabolites, and impurities) and activity data for six genotoxicity test types commonly employed in regulatory toxicology: the Ames test, the in vitro mammalian cell gene mutation test, the in vitro micronucleus test, the in vitro chromosomal aberration test, the in vivo micronucleus test, and the in vivo chromosomal aberration test. After merging and performing structure standardization on 18 public pesticide/biocide databases, we identified 4826 unique substances. Within 19 public genotoxicity databases, 19,897 substances had at least one data point in at least one genotoxicity test. The chemical space overlap between the pesticide substances and each genotoxicity set was evaluated by calculating physicochemical descriptors and molecular fingerprints, which were visualized by using dimensionality reduction methods. The chemical space of pesticide substances is well represented by substances with Ames test data and, to varying degrees, by substances with data from the other genotoxicity tests, with particularly low coverage for in vivo chromosomal aberration. The major scaffolds identified in pesticide substances were present in all of the genotoxicity data sets. Compared to pesticide substances, the genotoxicity data sets were enriched in functional groups characteristic of genotoxic compounds, such as annulated rings, but depleted in pesticide-typical structural motifs like halogens. Chemical space methods can assist regulatory toxicologists in understanding regions of pesticide substance chemical space that are well- or poorly characterized by genotoxicity data. This understanding is important for the accurate and targeted use of databases and data-based nontesting methods in line with regulatory requirements.

## Graphical abstract

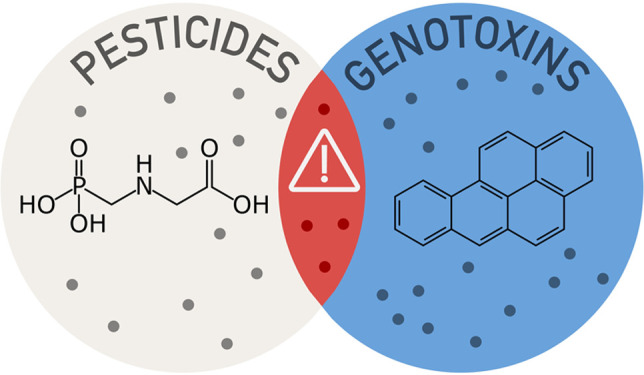

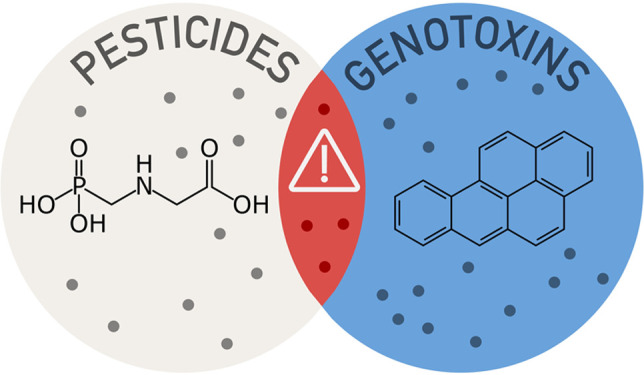

## Introduction

Understanding the potential of small molecules to cause genotoxicity is crucial to the safe use of these chemicals, ensuring the protection of human health and the environment. In the European Union, plant protection products^1^ and biocides^2^ (hereafter collectively referred to as pesticides) are subject to strict genotoxicity test requirements before authorization for use. Similar data requirements are in force in the USA,^3^ Japan,^4^ and China.^5^ The assessment typically involves a core battery of in vitro tests covering the apical end points of gene mutation and chromosome damage, with follow-up in vivo testing when indicated due to positive in vitro results.

The standard in vitro battery comprises the bacterial reverse mutation test (Ames test; OECD Test Guideline (TG) 471),^6^ a mammalian cell gene mutation assay (MCGM; OECD TG 476),^7^ and a chromosome damage assay, most commonly the in vitro micronucleus (MN) test (OECD TG 487),^8^ which detects both clastogenicity (structural chromosome damage) and aneugenicity (abnormal number of chromosomes). The in vitro chromosomal aberration (CA) test (OECD TG 473)^9^ is an alternative, but it is used less frequently. Where follow-up is needed, e.g., for pesticidal active substances or in the case of an in vitro positive, in vivo assays such as the mammalian erythrocyte micronucleus test (OECD TG 474)^10^ or bone marrow chromosomal aberration test (OECD TG 475)^11^ provide additional information. These internationally agreed-upon test methods form the regulatory basis for assessing the genotoxic potential of pesticides.

Although genotoxicity data for pesticides are publicly disclosed during the regulatory approval process, testing data for pesticide metabolites and impurities often remain confidential, hindering a broader understanding of the possible genotoxic activity of these substances and limiting the application of nontesting approaches such as QSAR and read-across. In contrast, a wealth of genotoxicity test data on small molecules is available in the public domain; however, these databases do not focus on pesticides. Instead, these databases typically result from broad literature searches for any substance with genotoxicity test data. The resulting data sets include substances from pharmaceutical research and development as well as prototypical genotoxicants used to investigate the fundamental mechanisms of genotoxicity. However, the overlap of these data sets with pesticide-relevant chemicals, including active substances, metabolites, and impurities, is unclear.

Nontesting approaches are gaining importance within emerging OECD and EFSA frameworks to reduce animal testing and accelerate the evaluation of chemicals. However, their reliability depends on the availability of structurally relevant, well-characterized reference compounds. If public databases lack adequate coverage of the pesticide chemical space, their utility for regulatory purposes will be limited.

In this work, we study the coverage of the pesticide chemical space with public genotoxicity data. More specifically, we investigate the physicochemical properties, Murcko scaffolds^12^ (i.e., the core molecular frameworks with peripheral substituents removed), and functional groups of pesticides over- or underrepresented in the public genotoxicity data. By mapping pesticide data sets from regulatory authorities against public genotoxicity data for the apical endpoints mutagenicity and chromosome damage, we highlight both areas of alignment and regions where structural coverage is limited. To provide a broader context, we included DrugBank^13^ as a reference data set to illustrate how the chemical space of pesticides and genotoxicity test data compares to the drug-like chemical space. We expect that our findings will support informed decisions on the applicability of read-across, QSAR, and other nontesting approaches and help guide future efforts to strengthen the regulatory data landscape for pesticide genotoxicity.

## Methods

Figure 1 summarizes the workflow employed here, including data collection, data preprocessing, data organization, and chemical space analysis.

**Figure 1:**
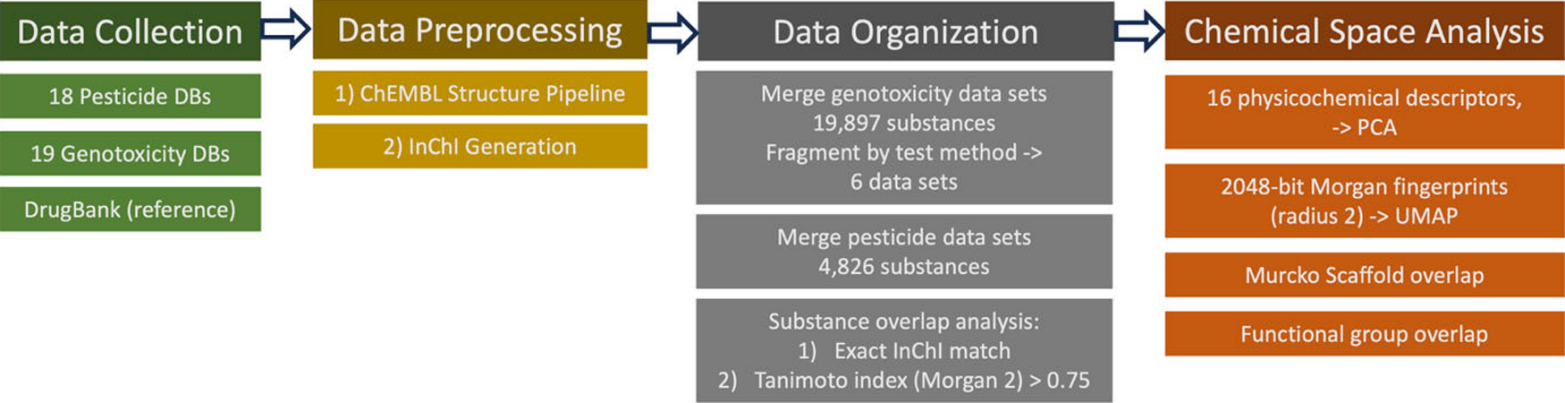
Flowchart depicting the workflow. A total of 19 public genotoxicity and 18 public pesticide data sets were collected, and DrugBank was also included as a reference set. The chemical structures were standardized with the ChEMBL Structure Pipeline, and InChIs were generated. The pesticide data sets were merged together, and the genotoxicity data sets were collated into one data set for each of the six genotoxicity test types. The overlap of the pesticide data set with each genotoxicity data set was computed in two ways: (1) exact match of InChIs; (2) considering two molecules from different data sets to be similar if their Tanimoto index (for the Morgan 2 fingerprint was >0.75). Then the chemical space overlap was visualized, and the coverage of pesticide Murcko scaffold and functional groups by the end point genotoxicity data sets was determined.

### Data Sets

Data sets on pesticide substances (i.e., active substances, metabolites, and impurities) and genotoxicity were collated from various public sources (Tables 1 and 2). A narrative description of the pesticide and genotoxicity data sets is provided below. In addition, data on approved and investigational drugs were retrieved from DrugBank^13^ (version 5.1.13).^14^ DrugBank was incorporated as a reference data set to represent the chemical space of pharmaceutically relevant compounds. This data set was included to enable comparison of pesticide chemical space with drug-like chemical space and to facilitate evaluation of whether the genotoxicity databases display biases toward pharmacologically relevant compounds versus agrochemicals. (Information about the Murcko scaffolds and the number of substances in the DrugBank sets is presented in Table S1).

**Table 1:** Pesticides Data Set Summary

data set	# substances (initial data set)^a^	# substances (curated data set)^b^	# exclusive substances^c^	# Murcko scaffolds^d^	mean molecules per Murcko (SD)^e^	# Murcko singletons (singletons per scaffold)^f^	mean pairwise Tanimoto coefficient^g^	description	reference
BfR	1689	1573	472	568	2.39 (12.60)	413 (0.73)	0.0936	active substances, metabolites and impurities of plant protection products and biocides	^15,16^
CP_DAT	414	355	73	98	2.28 (8.33)	78 (0.80)	0.0801	chemicals in consumer products; only pesticide-relevant subset was used	^17,19–27^
CompTox_Actives	488	474	1	250	1.62 (3.89)	207 (0.83)	0.0985	active substances - CompTox list: PESTICIDES|EPA	^28^
EFSA_PARAM	1079	1063	116	385	2.42 (11.68)	281 (0.73)	0.0993	plant protection product (PPP) subset of the EFSA PARAM Catalogue	^29^
EFSA_PestGentox	738	702	181	260	2.49 (7.60)	169 (0.65)	0.108	active substances and metabolites	^30,31^
EPAOPPIN	3382	2668	97	695	2.75 (20.95)	500 (0.72)	0.0818	substances related to pesticides according to the USEPA Office of Pesticide Programs	^32^
EPAPCS	3338	2649	70	679	2.81 (21.05)	482 (0.71)	0.082	active substances of pesticides according to the USEPA Office of Pesticide Programs	^33^
EUBIOCIDES	143	136	6	63	1.68 (2.21)	47 (0.75)	0.0819	compounds currently used (or recently banned) in the EU as biocides	^35^
FLUOROPEST	322	319	45	168	1.83 (2.68)	127 (0.76)	0.147	fungicides, herbicides, and insecticides with fluoro-moieties	^36,37^
LUXPEST	386	384	8	199	1.73 (3.68)	156 (0.78)	0.1039	pesticides and transformation products relevant for Luxembourg	^38,39^
NDATAPEST	832	829	57	369	2.06 (8.22)	284 (0.77)	0.1124	pesticide residue substances found in fruit and vegetables	^40,41^
NPINSECT	84	84	77	68	1.22 (0.73)	59 (0.87)	0.1232	natural product insecticides	^42^
PESTHHBS	428	409	0	236	1.56 (3.26)	194 (0.82)	0.1063	pesticide substances present in groundwater	^43,44^
PPDB	1468	1376	138	507	2.27 (10.88)	372 (0.73)	0.0969	pesticides properties database of the University of Hertfordshire	^45,46^
SLUPESTTPS	391	390	51	142	2.64 (6.92)	76 (0.54)	0.1105	suspect list of pesticides and pesticide transformation products	^47,48^
SWISSPEST	182	181	0	108	1.50 (1.85)	88 (0.81)	0.1001	insecticides, herbicides, and their transformation products	^49,50^
SWISSPEST19	876	876	210	257	3.02 (14.32)	162 (0.63)	0.0999	plant protection products and their transformation products	^51,52^
UBABIOCIDES	50	48	0	30	1.53 (1.25)	23 (0.77)	0.1127	biocide active substances and transformation products	^53^
total	NA	4826	NA	1190	3.21 (28.55)	791 (0.66)	0.0854		

a Number of unique SMILES strings in the original data set.

b Number of unique InChI strings in the data set after curation with the ChEMBL structure pipeline.

c Number of unique InChI strings occurring solely in a given data set.

d Number of unique Murcko scaffolds.

e Mean number of molecules per Murcko scaffold with standard deviation in parentheses.

f Number of Murcko singletons (scaffolds with only one member), with the number of singletons divided by the number of scaffolds in parentheses.

g Mean pairwise Tanimoto coefficients within the data set (derived from Morgan fingerprints with a radius of 2 and a length of 2048 bits).

**Table 2:** Genotoxicity Data Set Summary

data set	# substances (initial data set)^a^	# substances (curated data set)^b^	# exclusive substances^c^	overlap with pesticide substances^d^	overlap with approved drugs of DrugBank^e^	overlap with DrugBank (all) substances^f^	# Murcko scaffolds^g^	mean molecules per Murcko (SD)^h^	# Murcko singletons (singletons per scaffold)^i^	mean pairwise Tanimoto coefficient^j^	description	assay type(s)^k^	Reference
AMINES	622	617	147	20 (3%)	7 (1%)	23 (4%)	170	3.63 (14.14)	104 (0.61)	0.161	QSAR model data set - specific model for amines	Ames	^54,55^
AmesFormer	10,377	9693	2177	796 (8%)	392 (4%)	780 (8%)	2287	3.52 (31.63)	1363 (0.60)	0.082	QSAR model data set	Ames	^57,58^
Benfenati	5768	5762	34	555 (10%)	218 (4%)	470 (8%)	1337	3.57 (28.39)	912 (0.68)	0.0877	QSAR model data set	Ames	^59,60^
BfR	449	422	231	422 (100%)	4 (1%)	19 (5%)	158	2.49 (8.29)	108 (0.68)	0.1122	genotoxicity data for pesticide active substances, impurities, and metabolites	Ames, in vitro MN	^15,16^
CAESAR	4204	4204	0	496 (12%)	213 (5%)	448 (11%)	1070	3.26 (23.35)	757 (0.71)	0.087	QSAR model data set	Ames	^61,62^
CORAL	477	477	0	158 (33%)	71 (15%)	119 (25%)	139	2.47 (11.18)	113 (0.81)	0.0812	QSAR model data set	in vitro CA	^63,64^
ECVAMneg	208	199	0	97 (49%)	42 (21%)	70 (35%)	89	1.62 (4.05)	76 (0.85)	0.0755	multiassay data set for validation of new genotoxicity methods	Ames, in vitro CA, in vitro MCGM, in vitro MN, in vivo CA, in vivo MN	^65,66^
ECVAMpos	677	651	26	133 (20%)	63 (10%)	102 (16%)	201	2.44 (8.87)	146 (0.73)	0.0778	multiassay data set for validation of new genotoxicity methods	Ames, in vitro CA, in vitro MCGM, in vitro MN, in vivo CA, in vivo MN	^67–69^
EFSA_PestGentox	738	697	76	697 (100%)	13 (2%)	41 (6%)	260	2.48 (7.54)	169 (0.65)	0.1085	genotoxicity data on pesticide active substances and metabolites	Ames, in vitro CA, in vitro MCGM, in vitro MN, in vivo CA, in vivo MN	^30,31^
Hansen	6512	6504	0	591 (9%)	273 (4%)	578 (9%)	1547	3.48 (27.99)	1050 (0.68)	0.0854	literature data set	Ames	^70^
IRFMN	1218	1209	73	533 (44%)	108 (9%)	202 (17%)	460	2.07 (10.39)	366 (0.80)	0.0779	QSAR model data set	in vivo MN	^71,72^
ISS	666	664	12	229 (34%)	89 (13%)	134 (20%)	214	2.20 (9.73)	167 (0.78)	0.0717	QSAR model data set	Ames	^73,74^
ISSMIC	556	270	47	84 (31%)	37 (14%)	65 (24%)	112	1.66 (4.40)	95 (0.85)	0.0708	QSAR-ready data set	in vivo MN	^75,76^
ISSSTY	6759	1558	105	192 (12%)	66 (4%)	136 (9%)	452	3.14 (13.88)	320 (0.71)	0.1217	QSAR-ready data set	Ames	^77,78^
Karamertzanis	12,501	12,398	4991	1460 (12%)	367 (3%)	812 (7%)	2637	3.61 (40.34)	1795 (0.68)	0.0806	QSAR model data set	Ames, in vitro CA, in vitro MCGM, in vitro MN	^79^
OCHEM	11,506	9720	2235	878 (9%)	684 (7%)	1,130 (12%)	2495	3.26 (28.05)	1590 (0.64)	0.0816	literature data set	Ames	^80,81^
SARPY	4204	4204	0	496 (12%)	213 (5%)	448 (11%)	1070	3.26 (23.35)	757 (0.71)	0.087	QSAR model data set	Ames	^82,83^
VERMEER	379	376	11	90 (24%)	52 (14%)	84 (22%)	148	1.86 (6.63)	123 (0.83)	0.0836	QSAR model data set	in vitro MN	^84,85^
Xu	8408	7333	130	677 (9%)	313 (4%)	642 (9%)	1599	3.80 (29.47)	942 (0.59)	0.0844	literature data set	Ames	^86^
Total		19,897		2096 (11%)	804 (4%)	1480 (7%)	4554	3.52 (42.79)	2776 (0.61)	0.0795			

a Number of unique SMILES strings in the original data set.

b Number of unique InChI strings in the data set after curation with the ChEMBL structure pipeline.

c Number of unique InChI strings occurring solely in a given data set.

d Number of substances in the data set exactly matching an InChI present in the pesticides, Total data set.

e Number of substances in the data set exactly matching an InChI present in the approved drugs subset of DrugBank.

f Number of substances in the data set exactly matching an InChI present in the set of all DrugBank substances.

g Number of unique Murcko scaffolds.

h Mean number of molecules per Murcko scaffold with standard deviation in parentheses.

i Number of Murcko singletons (scaffolds with only one member), with the number of singletons divided by the number of scaffolds in parentheses.

j Mean pairwise Tanimoto coefficients within the data set (derived from Morgan fingerprints with a radius of 2 and a length of 2048 bits).

#### Pesticide Data Sets

All pesticide data sets are publicly available and are listed in Table 1.

The BfR data set, published by the German Federal Institute for Risk Assessment (Bundesinstitut für Risikobewertung, BfR), comprises active substances, impurities, and metabolites of plant protection products and biocides.^15,16^ The BfR data set also includes genotoxicity test data (i.e., Ames and in vitro micronucleus assay data) for a subset of substances.

The CP_DAT data set is derived from the Chemical and Products Database of the United States Environmental Protection Agency (USEPA), a comprehensive collection of information characterizing chemicals and their usage in commerce.^17^ The full CP_DAT database contains information about various types of chemicals and their specific uses, such as cosmetics, food contact materials, battery components, and adhesives. To include only relevant substances, only Product Use Categories^18^ of CP_DAT relevant to pesticides were included in this work: Landscape/yard: herbicide,^19^ Pesticides: fungicide,^20^ Pesticides: insecticide,^21^ Pet and animal care: all pets: pesticide,^22^ Pesticides: Professional use pesticides,^23^ Pesticides: animal repellant,^24^ Pesticides: insect repellant,^25^ Pesticides: rodenticide,^26^ and Pesticides: insect repellant - skin.^27^

The CompTox_Actives data set is derived from USEPA’s list of pesticide active ingredients, retrieved from CompTox Chemicals Dashboard.^28^ The EFSA (European Food Safety Authority) PARAM, part of EFSA’s Data Collection Framework, a harmonized terminology collection developed to support scientific research, is a catalog that contains a list of analytically detectable substances which may be found in food or feed.^29^ The subset used here was filtered to only keep substances marked as “PPP”, indicating that they are active substances, impurities, or metabolites of plant protection products. Published by EFSA, the EFSA_PestGentox data set contains genotoxicity data for pesticide active substances and metabolites, compiled from regulatory submissions.^30,31^ The EPAOPPIN data set is derived from the integrated database for regulatory and scientific data of the USEPA Office of Pesticide Programs Information Network (OPPIN), and contains information on pesticide active substances and metabolites.^32^ The EPAPCS data set is sourced from the USEPA’s Pesticide Chemical Search database and contains pesticide active substances.^33^ The EUBIOCIDE data set is hosted by the NORMAN Suspect List Exchange (NORMAN-SLE)^34^ and comprises substances that are either currently used in the EU as biocides or those that have been recently banned.^35^ The FLUOROPEST data set, hosted by NORMAN-SLE, describes fluorinated insecticides, herbicides, and fungicides.^36,37^ Describing pesticides and their transformation products relevant for Luxembourg, LUXPEST is hosted by NORMAN-SLE.^38,39^ The NDATAPEST data set comprises pesticide residues found in fruit and vegetables.^40,41^ Hosted by NORMAN-SLE, the NPINSECT data set comprises natural product insecticides.^42^ The PESTHBBS data set from the USEPA describes pesticide residues found in drinking water, together with data on reference doses for acute and chronic toxicity.^43,44^ The PPDB data set represents the Pesticides Properties Database of the University of Hertfordshire, hosted by NORMAN-SLE.^45,46^ Created based on Sweden’s national pesticide monitoring program, SLUPESTTPS contains pesticides and pesticide transformation products, and is hosted by NORMAN-SLE.^47,48^ SWISSPEST^49,50^ and SWISSPEST19 ^51,52^ represent insecticides, fungicides, and their transformation products relevant for Switzerland, and are hosted by NORMAN-SLE. UBABIOCIDES—developed by the German Environment Agency (UBA)—is hosted by NORMAN-SLE and comprises a list of prioritized biocide active substances and transformation products.^53^

#### Genotoxicity Data Sets

All genotoxicity data sets are publicly available and are listed in Table 2. The AMINES data set is derived from an Amines-specific QSAR model for the Ames test,^54,55^ available through the VEGA platform.^56^ The AmesFormer data set represents the publicly available portion of the training set for the Ames QSAR model of the same name.^57,58^ The Benfenati data set is derived from a VEGA QSAR model for Ames referred to as Mutagenicity Model KNN-Read-across.^59,60^ The BfR data set, containing Ames and in vitro micronucleus test results for active substances, impurities, and metabolites of plant protection products and biocides, was developed by the German Federal Institute for Risk Assessment.^15,16^ The CAESAR data set constitutes an additional VEGA data set for Ames mutagenicity.^61,62^ The CORAL data set was derived from a VEGA QSAR model for in vitro chromosomal aberration.^63,64^ The ECVAMneg^65,66^ and ECVMApos^67–69^ data sets are multi-end point data sets originally developed by the European Centre for the Validation of Alternative Methods in order to support the validation of new genotoxicity test methods. They contain data for the Ames test, in vitro chromosomal aberration test, in vitro Mammalian Cell Gene Mutation test, in vitro micronucleus test, in vivo chromosomal aberration test, and in vivo micronucleus test. The ECVAMneg set focuses on substances that are negative in the Ames test, while the ECVAMpos set focuses on Ames positives. The EFSA_PestGentox data set contains genotoxicity testing data on pesticide active substances and metabolites from regulatory submissions to EFSA or European member states during the pesticide approval/renewal process.^30,31^ All genotoxicity test types included in this analysis are listed in the EFSA_PestGentox data set. The Hansen data set was compiled by gathering Ames test data from various databases and literature sources.^70^ The IRFMN data set is from a VEGA QSAR model for the in vivo micronucleus test.^71,72^ The ISS data set was obtained from VEGA and comprises the database used to generate the Benigni/Bossa ruleset for the Ames mutagenicity test.^73,74^ Described by its creators as a “QSAR-ready” data set, the ISSMIC data set contains in vivo micronucleus test data and was published by the Italian health authority ISS.^75,76^ Another ISS QSAR-ready data set is the ISSTY data set, containing Ames test data.^77,78^ The Karamertzanis data set resulted from a collaboration between the USEPA and ECHA, and comprises data on a variety of in vitro genotoxicity test methods (Ames, in vitro CA, in vitro MCGM, and in vitro MN) derived from REACH and other public data sources.^79^ The OCHEM data set contains Ames test data and was retrieved from the Online Chemical Modeling Environment (OCHEM).^80,81^ The SARPY data set represents yet another VEGA QSAR model training data set for Ames mutagenicity.^82,83^ The VERMEER data set is derived from a VEGA QSAR model for in vitro micronucleus.^84,85^ The Xu data set is a literature data set for the Ames mutagenicity test.^86^

### Data Preprocessing

Given the variety of data sources, curation and standardization of the chemical structures were necessary. For each chemical entity, the SMILES string^87^ was validated and standardized using the “standardize” and “get_parent” functions of ChEMBL Structure Pipeline^88^ (version 1.2.0).^89^ The “standardize” function includes several steps, the most relevant of which are summarized here: (1) standardize unknown stereochemistry; (2) Kekulize the structure; (3) remove hydrogen atoms; (4) normalize the charged substructures so that they are depicted consistently; (5) neutralize the molecule (with a list of defined neutralization rules); and (6) normalize triple bonds. In a second step, the get_parent function performs several operations: (1) set all isotopes to 0 and remove any hydrogen atoms not previously removed (i.e., D and T atoms); (2) remove all solvents (defined in a list) unless doing so removes all fragments; (3) remove salt components (defined in a list) unless doing so removes all fragments; (4) remove duplicate fragments (fragments with different stereochemistry are considered nonduplicates); and (5) neutralize the remaining molecule if possible.

Furthermore, any SMILES that failed any standardization step were excluded from the analysis. Similarly, SMILES strings containing the wild-card character (*) were also discarded, as they could not be assigned a specific chemical structure. These wildcard substances were exclusively substances of variable composition originating from USEPA’s CompTox Chemicals Dashboard and are listed in Table S2. The tautomeric forms and stereochemical information provided with the original chemical information were maintained. Using RDKit (version 2023.9.5),^90^ InChI were generated from the standardized molecules.

The standardization and canonicalization process reduced the number of unique molecules (represented by unique InChI strings) from 54,343 to 22,627. Of these 22,627 compounds, 19,842 have data in at least one of the genotoxicity study types, and 4826 are present in one or more of the pesticide data sets. Molecules were considered identical across two data sets if they had identical InChI and exclusive if they occurred only in a single data set.

When integrating the genotoxicity data from separate sources, it is desirable to reach an overall conclusion regarding the activity (hit call) for each substance in each assay. Since the focus of this work is the genotoxicity of pesticides in a regulatory context, conservative criteria were considered appropriate: a substance was only called “negative” if it was unanimously “negative” in a given assay; any substance which had one or more “positive” results was called “positive”, regardless of the number of “negative” results in the data set. Thus, the entirety of the genotoxicity was considered, and only substances with exclusively negative test results were considered negative. In contrast, a positive result in one or more genotoxicity tests was considered to be positive. It should be noted that this analysis did not assess the quality or regulatory compliance of the individual genotoxicity studies (e.g., adherence to the OECD test guidelines or GLP standards). While such an evaluation was beyond the scope of the present work, study quality is an important consideration in regulatory decision-making, as non-OECD or non-GLP results may carry a different interpretive weight than fully compliant studies.

### Overlap and Similarity Calculation

To determine the overlap between each genotoxicity end point data set (i.e., a single collated data set for each of the six genotoxicity test types) and the pesticides, the InChI string for each substance was used as a unique identifier. For each overlap analysis, the number of substances with matching InChI strings in both data sets was taken to be the overlap. The same approach was taken to compare each genotoxicity and end point data set to both the approved drugs subset of DrugBank as well as the entire DrugBank (i.e., also including investigational drugs). In a further step, the overlap of the genotoxicity and end point data sets with “similar” substances was checked: the Tanimoto similarity of Morgan fingerprints (radius of 2, 2048 bits) for the molecules in the pesticides and DrugBank sets with that of the molecules in each genotoxicity or end point data set was computed using FPSim2.^91^ Molecules were considered similar if their Tanimoto similarity was at least 0.75, a conservative choice stemming from the regulatory toxicology focus of this work.

### Calculation of Molecular Properties and Clustering of Molecules

Physicochemical descriptors, circular molecular fingerprints, and Murcko scaffolds for each molecule were calculated with RDKit (version 2023.9.5).^90^ Specifically, physicochemical descriptors calculated were molecular weight (MW), octanol/water partition coefficient (log*P*, calculated by the Crippen method), topological polar surface area (TPSA), hydrogen bond donor count, hydrogen bond acceptor count, heavy atom count, nitrogen count, oxygen count, sulfur count, halogen count, bridgehead atom count, chiral center count, aromatic atom count, total rings count, fraction sp^3^-hybridized carbons, and formal charge. Morgan fingerprints (2048-bit) with a radius of 2 were also calculated by using RDKit.

Murcko scaffolds represent the core ring systems of molecules after removal of all side chains. This process captures the fundamental structural backbone that contributes to—but does not solely determine—the biological properties of a molecule, which can also be influenced by specific functional groups or substituents. Data sets with a lower number of molecules per Murcko scaffold may be more chemically diverse than those with higher numbers of molecules per Murcko scaffold. Murcko scaffold singletons are molecules that exhibit a unique scaffold that is not present in any other molecule in the same data set. Lower singleton frequency indicates that a data set may be less chemically diverse than data sets with a higher singleton frequency. A particularly high singleton frequency may indicate that a data set may be difficult to model, as each singleton could represent a molecule on which there are no similar structures on which to train the model.

The presence or absence of functional groups in each molecule was checked against a list of SMARTS patterns^92^ extracted from Open Babel.^93^ The list of functional groups was filtered to remove any functional groups that did not occur in either the pesticide or genotoxicity data sets: of 305 SMARTS patterns from Open Babel, 266 were included in the analysis.

In order to visualize the molecular descriptors in human-readable two-dimensional images, two complementary dimensionality reduction techniques were used: Principal Component Analysis (PCA) for the physicochemical descriptors, and Uniform Manifold Approximation and Projection (UMAP)^94^ for the Morgan 2 fingerprints. The PCA was computed on the 16 physicochemical descriptors with Scikit-learn^95^ version 1.42,^96^ and the first two principal components were plotted. To motivate our selection of parameters for the UMAP, we first performed Butina clustering^97^ (cutoff = 0.5) of the Morgan 2 fingerprints with RDKit. The UMAP projections were calculated with the UMAP Python package (umap-learn version 0.5.5).^98^

A grid search of the UMAP parameters n_neighbors and min_dist was performed. n_neighbors controls the local neighborhood size used to build the manifold graph representation (balancing local versus global structure preservation), and min_dist determines the minimum allowable distance between points in the low-dimensional embedding (controlling the tightness of clustering in the final projection).

Trustworthiness,^99^ a measure of how similar (or dissimilar) two clustering outputs are to each other, was used to evaluate which UMAP clustering was most well aligned with the Butina clustering. For each pair of UMAP parameters, the Trustworthiness against Butina clustering was computed, and the UMAP parameters with the highest trustworthiness were selected. The results of the grid search, as well as the sensitivity analysis for each UMAP parameter, are shown in Figure S1.

PCA and UMAP offer complementary perspectives on the data. PCA provides a linear projection that captures the main axes of variance, making it well-suited for physicochemical descriptors, where a few components explain a large proportion of the total variance. However, PCA performs poorly on molecular fingerprints, where the explained variance is low and the data structure is highly nonlinear. Therefore, we used UMAP as an alternative approach for visualizing the fingerprint space as it better preserves both local and global similarity relationships among molecules. Because UMAP was applied to Morgan fingerprints, which numerically encode the presence and arrangement of substructural features, molecules with similar chemical scaffolds or functional groups are expected to be positioned near one another in the low-dimensional space. The resulting clusters thus reflect genuine chemical similarity rather than random proximity, allowing meaningful visualization of structurally related compounds.

## Results and Discussion

A total of 4826 unique pesticide substances were collected from 18 sources, representing 1190 unique Murcko scaffolds (Table 1). The largest data sets, EPAOPPIN^32^ and EPAPCS,^33^ contain 2668 and 2649 substances, respectively. BfR^16^ contributes the most exclusive entries (472), followed by SWISSPEST19 ^52^ (210). Smaller data sets focus on specific use cases, such as biocides (EUBIOCIDES,^35^ UBABIOCIDES)^53^ or insecticides (NPINSECT),^42^ particular compound classes like halogens (FLUOROPEST)^37^ or natural products (NPINSECT), or pesticides regulated by national jurisdictions (LUXPEST,^38^ SLUPESTTPS,^48^ SWISSPEST19). All pesticide data sets exhibit high intradata set diversity, as demonstrated by their low mean pairwise Tanimoto similarities, ranging from 0.147 (FLUOROPEST) to 0.0801 (CP_DAT). As shown in Figure 2, there is a linear relationship between the number of unique compounds and the number of unique Murcko scaffolds for the data sets under investigation. Further data on molecular diversity and other properties are provided in Tables 1 and 3.

**Table 3:** Per End Point Substance Summary

data set	# substances (curated data set)^a^	# exclusive substances^b^	overlap with pesticide substances^c^	overlap with pesticide substances similarity >0.75^d^	overlap with approved drugs of DrugBank^e^	overlap with approved drugs of DrugBank similarity >0.75^f^	overlap with DrugBank (All) substances^g^	overlap with DrugBank (All) substances similarity >0.75^h^	# Murcko scaffolds^i^	mean molecules per Murcko (SD)^j^	# Murcko singletons (singletons per scaffold)^k^	mean pairwise Tanimoto coefficient^l^	assay readouts^m^	
													positive	negative
**Ames**	18,945	13,677	1974 (10%)	2607 (14%)	799 (4%)	1683 (9%)	1439 (8%)	2899 (15%)	4388	3.51 (41.86)	2677 (0.61)	0.0799	7683	11,262
in vitro **MCGM**	3312	107	955 (29%)	1148 (35%)	156 (5%)	293 (9%)	356 (11%)	626 (19%)	823	2.61 (18.04)	587 (0.71)	0.0778	748	2564
in vitro **MN**	1329	60	399 (30%)	457 (34%)	92 (7%)	176 (13%)	182 (14%)	319 (24%)	416	2.24 (12.24)	308 (0.74)	0.0761	527	802
in vivo **MN**	1452	121	636 (44%)	699 (48%)	134 (9%)	263 (18%)	254 (17%)	447 (31%)	523	2.18 (10.90)	375 (0.72)	0.0764	518	934
in vitro **CA**	3841	294	1045 (27%)	1252 (33%)	228 (6%)	420 (11%)	447 (12%)	804 (21%)	950	2.77 (21.88)	660 (0.69)	0.0766	1475	2366
in vivo **CA**	319	1	166 (52%)	177 (55%)	38 (12%)	53 (17%)	72 (23%)	95 (30%)	138	1.78 (4.01)	117 (0.85)	0.0781	166	153
all genotoxicity end points	19,897	NA	2096 (11%)	2793 (14%)	804 (4%)	1732 (9%)	1480 (7%)	3052 (15%)	4554	3.52 (42.79)	2776 (0.61)	0.0795	9043	10,854
pesticides	4826	2730	4826 (100%)	4826 (100%)	222 (5%)	369 (8%)	463 (10%)	769 (16%)	1190	3.21 (28.55)	791 (0.66)	0.0854	799	1297
total	22,627	NA	4826 (21%)	5523 (24%)	849 (4%)	1,853 (8%)	1602 (7%)	3351 (15%)	5070	3.60 (48.09)	3130 (0.62)	0.0798	9043	10,854

a Number of unique InChI strings in the data set after curation with the ChEMBL structure pipeline.

b Number of unique InChI strings occurring solely in a given data set.

c Number of substances in the data set exactly matching an InChI present in the pesticides, Total data set.

d Number of substances in the data set with >0.75 Tanimoto similarity (2048-bit Morgan 2) to any substance in the Total pesticides data set.

e Number of substances in the data set exactly matching an InChI present in the approved drugs subset of DrugBank.

f Number of substances in the data set with >0.75 Tanimoto similarity (2048-bit Morgan 2) to any substance in the approved drugs subset of DrugBank.

g Number of substances in the data set exactly matching an InChI present in the set of all DrugBank substances.

h Number of substances in the data set with >0.75 Tanimoto similarity (2048-bit Morgan 2) to any substance in the set of all DrugBank substances.

i Number of unique Murcko scaffolds.

j Mean number of molecules per Murcko scaffold with standard deviation in parentheses.

k Number of Murcko singletons (scaffolds with only one member), with the number of singletons divided by the number of scaffolds in parentheses;

l Mean pairwise Tanimoto coefficients within the data set (derived from Morgan fingerprints with a radius of 2 and a length of 2048 bits).

m Substance-level assay readouts according to the conservative criteria described in the Methods section.

**Figure 2:**
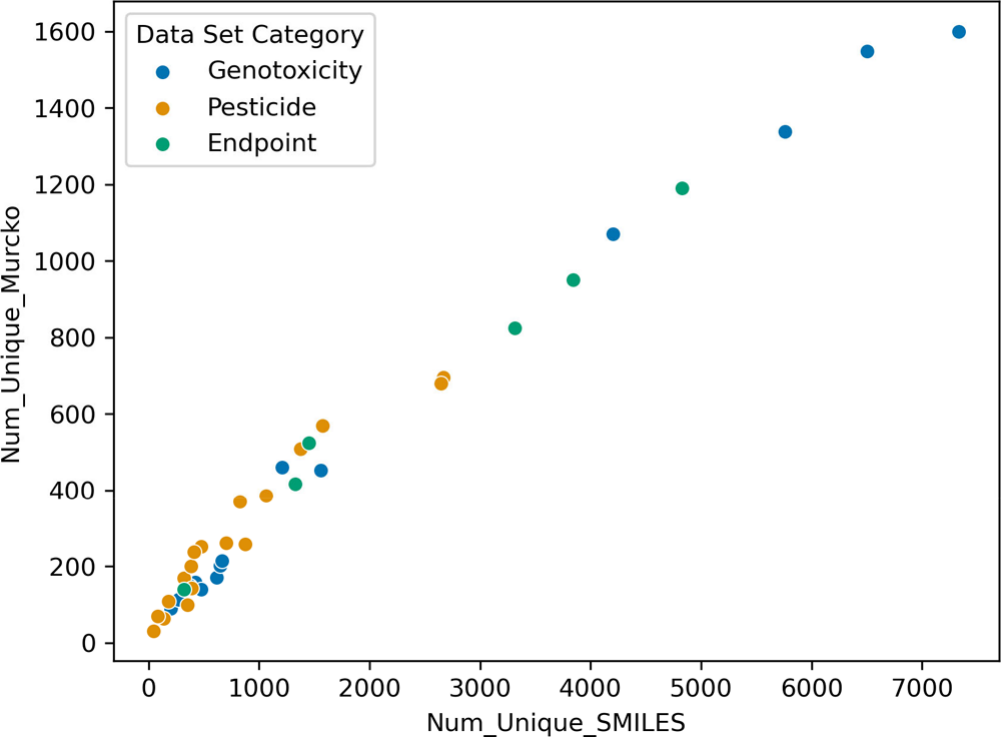
Scatterplot of unique Murcko scaffolds vs the number of SMILES data set. Chemical diversity, as measured by the number of unique Murcko scaffolds, scales similarly with data set size (i.e., number of unique SMILES) regardless of data set type. In this figure, the three data set types are the same as in Tables 1–3. Pesticide refers to the individual pesticide data sets in Table 1. Genotoxicity refers to the individual genotoxicity data sets in Table 2. End point refers to the merged data sets as presented in Table 3 (e.g., one data set for each genotoxicity test type as well as one for the merged set of pesticides). Two data sets lie beyond the axes and were excluded to enhance the visualization of the smaller data sets: the end point data set for Ames (18,945 unique SMILES, 4388 Murcko scaffolds), as well as the total set of substances with genotoxicity data (19,897 unique SMILES and 4554 Murcko scaffolds).

The genotoxicity data collected in this work comprise a total of 19,897 unique substances, representing 4544 Murcko scaffolds (Table 2). Among the 19 source data sets, the largest data set is the multiassay set from Karamertzanis et al., with 12,398 unique substances. The next largest data sets exclusively contain Ames test results, with the data set from OCHEM^80,81^ containing 9720 unique substances, AmesFormer^58^ counting 9693, and that of Xu et al.^86^ containing 7333. Karamertzanis et al.^79^ contribute the most exclusive substances (4991), followed by OCHEM (2235) and AmesFormer (2177). BfR^16^ has the highest proportional exclusivity (259 out of its 423 substances being exclusive), possibly reflecting the data source, which, to a large extent, was from regulatory dossiers for pesticides. As with the pesticides data sets, the genotoxicity data sets have high intradata set diversity, with mean pairwise Tanimoto similarities ranging from 0.161 (AMINES) to 0.0708 (ISSMIC). The relationship between the data set size and the number of Murcko scaffolds represented is consistent with that observed for the pesticide data sets under investigation (Figure 2).

When excluding the pesticide-specific genotoxicity data sets (BfR and EFSA_PestGentox), the proportion of each individual genotoxicity data set that overlaps with the collated set of pesticides varies from as little as 3% (AMINES)^54^ to as high as 49% (ECVAMneg).^65^ In total, 11% (2096 substances) of the genotoxicity data set also appear in pesticide data sets. Expanding the overlap criteria to include substances within a Tanimoto similarity of 0.75 (Table S3) only moderately increases the overlap to 4 to 55%, with 2793 (14%) genotoxicity substances meeting this similarity threshold with pesticides.

Similarly, the proportion of substances in each individual genotoxicity data set that are also approved drugs ranges from 1% (AMINES and BfR) to 21% (ECVAMneg).^65^ When including drugs within a 0.75 Tanimoto similarity (Table S3), the overlap increases across all data sets from 1 (BfR) to 31% (ECVAMneg). A total of 804 (4%) substances with genotoxicity data are approved drugs, while 1732 (9%) have high similarity (>0.75 Tanimoto) to an approved drug. Examining the broader drug-like chemical space, overlap with the full DrugBank ranges from 4 (AMINES) to 35% (ECVAMneg), with 1480 (7%) substances with genotoxicity data found in the complete DrugBank set. Expanding to include structurally similar substances (Tanimoto >0.75, Table S4) raises the overlap to 15% (3052 substances), varying between 7 (BfR) and 49% (ECVAMneg). Generally, genotoxicity data sets contain more pesticide substances than drugs, even when considering a broader definition of drug-like substances, i.e., including all substances in DrugBank.

Our analysis of combined genotoxicity and pesticide substances yielded 22,627 unique chemical entities, with 4826 classified as pesticide substances and 19,897 having data in at least one genotoxicity test (Table 3). Only 2096 substances (about 11% of all substances with genotoxicity data) overlap both categories, meaning 2730 pesticide substances lack genotoxicity testing data in these public data sets. The Ames test data set represents the most extensive collection, with 18,945 substances, and contains the most exclusive entries (13,677), while the in vivo CA data set is the smallest, with just 319 substances. Notably, the in vivo CA contains only one exclusive entry.

Pesticide substances constitute a substantial proportion (27 to 52%) of most of the genotoxicity test data sets examined, with the Ames test being the exception at only 10% (Table 3, data set “All Genotoxicity”). In contrast, approved drugs comprise a much smaller portion (4 to 12%) of genotoxicity data. However, this increases to 15 to 31% when including analogue substances within 0.75 Tanimoto similarity of any substance in DrugBank. While pesticide substances represent a substantial portion of genotoxicity data sets, the reverse relationship is weak. With the exception of the Ames test, public genotoxicity data sets cover only a small fraction of known pesticide substances, ranging from 22% coverage by the in vitro CA test (1045 of 4826) to merely 3% by the in vivo CA test (166 of 4826).

Again, the number of molecules representing a unique scaffold is lower for the smaller data sets than for the larger ones. The Ames data set has the highest number of molecules per scaffold (3.51), whereas the in vivo CA data set has both the lowest number of entries (319) and molecules per scaffold (1.78). The endpoint-level data sets all demonstrate high intradata set molecular diversity, as indicated by their low mean pairwise Tanimoto similarities (all <0.08).

The relationship between data set size and number of Murcko scaffolds displayed by the end point data sets followed the same linear relationship observed for the other types of data sets (Figure 2). Most end point data sets are biased toward negatives, likely due to the very large Karamertzanis et al. data set, which used data from REACH submissions. Since genotoxic substances are typically disallowed from use in commerce, it logically follows that the REACH data would be enriched with substances that have negative genotoxicity test results. Further information is provided in Table 3.

Table 4 presents the mean and standard deviation of 16 physicochemical properties for each end point. Pesticide substances show the highest mean molecular weight (283.62) and halogen count (1.09), as well as the second-highest mean log*P* (2.64). These higher log*P* values suggest that pesticide substances are, on average, more lipophilic than those in the end point data sets. Increased lipophilicity is associated with enhanced membrane permeability, potentially leading to greater bioaccumulation.^100,101^ The frequent presence of halogen atoms further raises the lipophilicity and can increase metabolic stability by reducing susceptibility to biotransformation,^102,103^ making these compounds more persistent in biological systems. Interestingly, pesticides also contain more chiral centers than the molecules represented in the genotoxicity data sets.

**Table 4:** Physicochemical Properties by End Point Data Set^a^

data set	MW	log*P*	TPSA	hydrogen bond acceptor count	hydrogen bond donor count	heavy atom count	nitrogen count	oxygen count
pesticides	283.62 (133.69)	2.64 (2.21)	59.34 (43.78)	3.67 (2.67)	1.05 (1.37)	18.32 (9.21)	1.42 (1.59)	2.81 (2.47)
Ames	279.92 (155.52)	2.67 (2.30)	62.47 (56.81)	3.72 (3.10)	1.21 (1.79)	19.19 (10.91)	1.50 (1.90)	2.80 (2.80)
in vitro MCGM	254.81 (145.55)	2.55 (2.58)	55.90 (46.15)	3.37 (2.61)	1.06 (1.34)	17.20 (9.91)	1.25 (1.66)	2.64 (2.30)
in vitro MN	240.72 (146.17)	2.19 (2.31)	57.32 (52.45)	3.32 (2.82)	1.15 (1.63)	16.26 (10.06)	1.27 (1.72)	2.59 (2.52)
in vivo MN	265.44 (166.92)	2.15 (2.27)	64.20 (59.88)	3.75 (3.35)	1.37 (1.92)	17.67 (11.44)	1.52 (1.82)	2.86 (3.08)
in vitro CA	255.10 (147.25)	2.49 (2.50)	56.50 (49.14)	3.33 (2.69)	1.11 (1.45)	17.14 (9.95)	1.25 (1.64)	2.63 (2.45)
in vivo CA	245.17 (150.49)	2.34 (2.23)	54.42 (49.33)	3.33 (2.89)	0.97 (1.57)	16.30 (10.49)	1.40 (1.69)	2.37 (2.45)

data set	sulfur count	halogen count	bridgehead atom count	chiral center count	aromatic atom count	total ring count	fraction sp^3^	formal charge
pesticides	0.34 (0.71)	1.09 (1.76)	0.08 (0.51)	0.46 (2.17)	6.14 (5.48)	1.57 (1.34)	0.43 (0.31)	0.02 (0.19)
Ames	0.21 (0.58)	0.57 (1.48)	0.06 (0.48)	0.42 (1.77)	7.63 (7.01)	2.09 (1.77)	0.40 (0.33)	0.01 (0.13)
in vitro MCGM	0.23 (0.62)	0.60 (1.61)	0.04 (0.33)	0.06 (0.57)	5.17 (6.28)	1.30 (1.33)	0.53 (0.35)	0.00 (0.05)
in vitro MN	0.20 (0.60)	0.49 (1.33)	0.04 (0.29)	0.10 (0.73)	5.36 (6.12)	1.43 (1.44)	0.47 (0.36)	0.01 (0.08)
in vivo MN	0.21 (0.56)	0.80 (1.77)	0.04 (0.33)	0.20 (1.00)	6.51 (6.20)	1.79 (1.78)	0.39 (0.32)	0.02 (0.20)
in vitro CA	0.22 (0.62)	0.70 (1.97)	0.04 (0.32)	0.08 (0.75)	5.50 (6.23)	1.38 (1.35)	0.49 (0.35)	0.00 (0.05)
in vivo CA	0.29 (0.66)	0.65 (1.31)	0.04 (0.37)	0.33 (1.31)	6.96 (6.07)	1.70 (1.45)	0.34 (0.32)	0.00 (0.19)

a Means (with standard deviations in parentheses) for the 16 physicochemical descriptors. The pesticides data set was compiled from 18 public pesticides data sets, whereas the other data sets result from the collation of 19 genotoxicity data sets, subsequently fragmented by genotoxicity test type.

The in vivo CA data set exhibits less complex molecules with the lowest hydrogen bond donor count (0.97) and greater planarity (lowest fraction of sp^3^ hybridized atoms (0.34)) compared to the Ames data set, which contains significantly more complex, aromatic-rich structures.

Two complementary methods were employed to analyze the coverage of pesticides with genotoxicity data. Principal Component Analysis (PCA) was applied to physicochemical descriptors, with the first two principal components accounting for 50.9% of the total variance (Figure 3). The loading plot (Figure 4) indicates that properties that scale with molecular size, such as molecular weight and atom counts, are key for PC1, while lipophilicity, aromaticity, and saturation play significant roles in PC2. Although some areas of physicochemical descriptor space lack genotoxicity test data for pesticide substances, many nonpesticide substances with such data are nearby. Notably, Ames data provides good coverage of the pesticide descriptor space, while other genotoxicity tests exhibit both well-covered and data-deficient regions. The in vivo CA data are the sparsest, revealing deficiencies in both large molecules (high MW, high atom count) and those with multiple rings and large numbers of aromatic atoms. A similar pattern can be observed in the other non-Ames data sets. From a regulatory perspective, these areas of physicochemical space could be targeted for future data gathering or generation activities when seeking to understand the genotoxic potential of pesticides as a whole. Furthermore, these regions of chemical space, without sufficient data, could help explain why QSAR models for non-Ames genotoxicity are not currently considered to have sufficient performance for standalone use in regulatory toxicology.^104^

**Figure 3:**
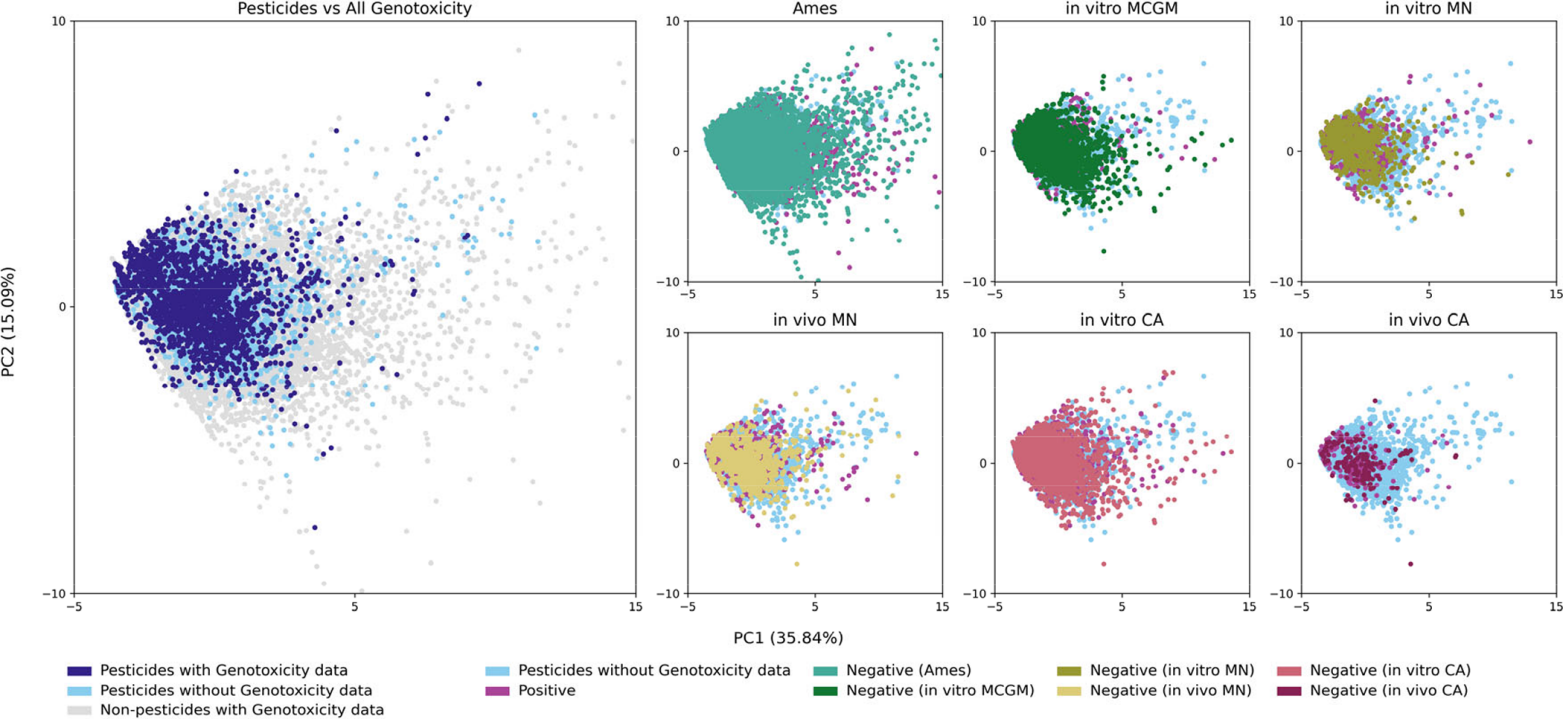
Scatter plots and loading plot of the first two principal components of a principal component analysis calculated on the 16 physicochemical descriptors, as detailed above. The *x*-axis of each subplot represents the first principal component (PC1), which describes 35.8% of the variance in the total data set. The second principal component (PC2), accounting for 15.1% of the variance, is represented on the *y*-axis. To aid in visualization, a small number of outliers (56 substances out of 22,627) that lie beyond the bounds of the plot are not displayed.

**Figure 4:**
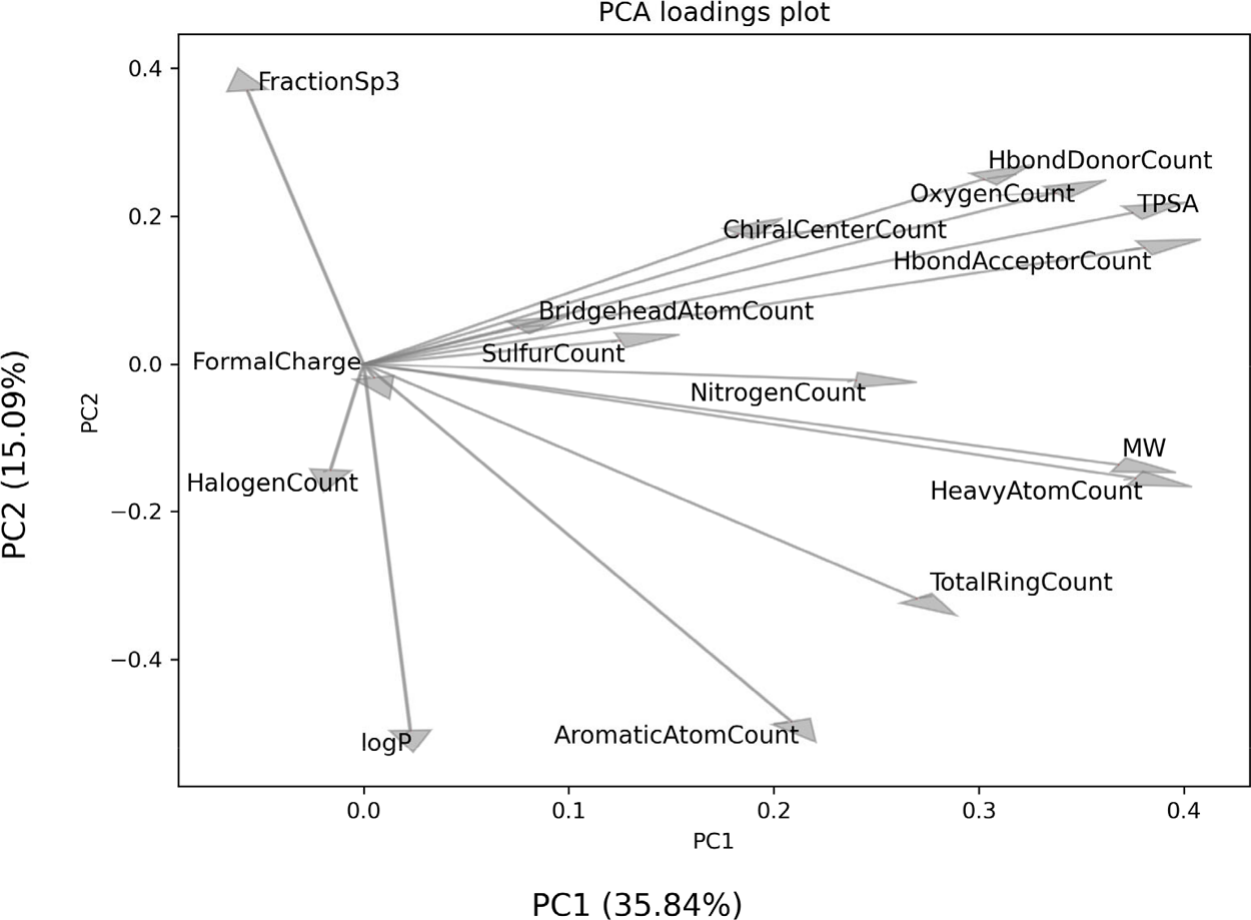
Loadings plot for the principal component analysis computed on the 16 physicochemical descriptors as shown in Figure 3. The descriptors most strongly associated with the first principal component (PC1) include those scaling with molecular size, such as MW, surface area, and atom counts. Lipophilicity and aromaticity are strongly correlated to the second principal component (PC2).

Uniform Manifold Approximation and Projection (UMAP) was applied to Morgan 2 fingerprints (Figure 5). UMAP parameters were min_distance set to 0.01 and n_neighbors set to 3 (see Methods for details on how UMAP parameters were selected). The analysis revealed that pesticide substances form distinct clusters, reflecting their structural diversity. While most clusters with pesticides contain at least one pesticide with genotoxicity data, several lack such data. Nonpesticide substances appear to be dispersed throughout but also form distinct clusters separate from pesticides, indicating structural differences. From a regulatory perspective, the presence of pesticide clusters with genotoxicity data provides confidence that structurally related compounds have at least some empirical evidence supporting their safety assessment via read-across or QSAR. Conversely, clusters lacking genotoxicity coverage represent structural families of pesticides for which regulatory agencies must currently rely on read-across, predictive models, or new testing. This gap is particularly notable in the case of the in vivo chromosomal aberration (CA) test, where several pesticide-rich clusters remain uncharacterized, underscoring a key area of toxicological uncertainty. In contrast, Ames, in vitro MCGM, and in vitro CA assays exhibit broader coverage, suggesting that the circular fingerprint chemical space of pesticides is well covered by these data.

**Figure 5:**
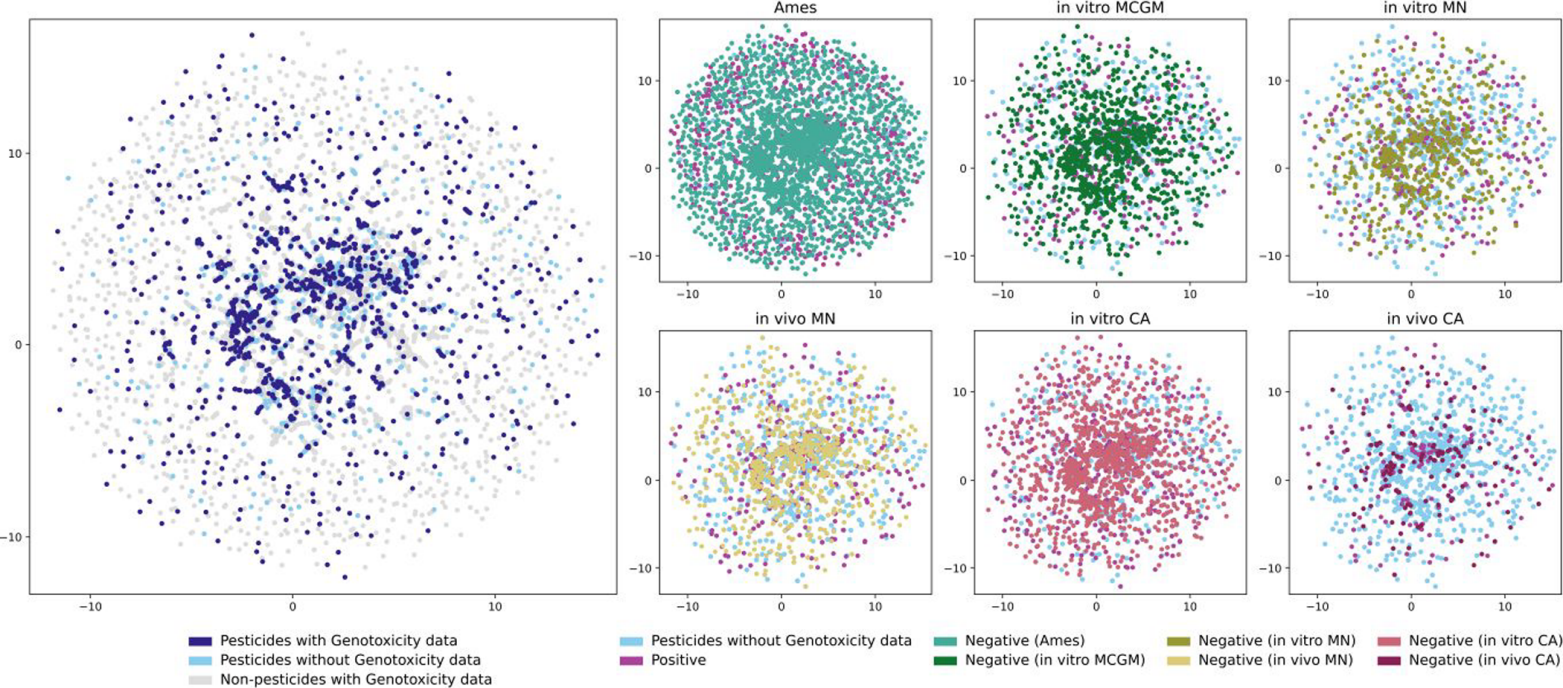
Scatter plot of the UMAP, showing how different genotoxicity data sets have different coverages of the pesticide-relevant chemical space.

Figure 6 shows that molecules with similar chemical structures cluster together, as evident in the magnified regions where compounds within each cluster share structural features or functional groups. The spatial separation between clusters (such as between regions A and D) represents distinct chemical spaces with different structural motifs, highlighting how UMAP captures meaningful chemical relationships by placing structurally related compounds in proximity while distancing chemically dissimilar ones.

**Figure 6:**
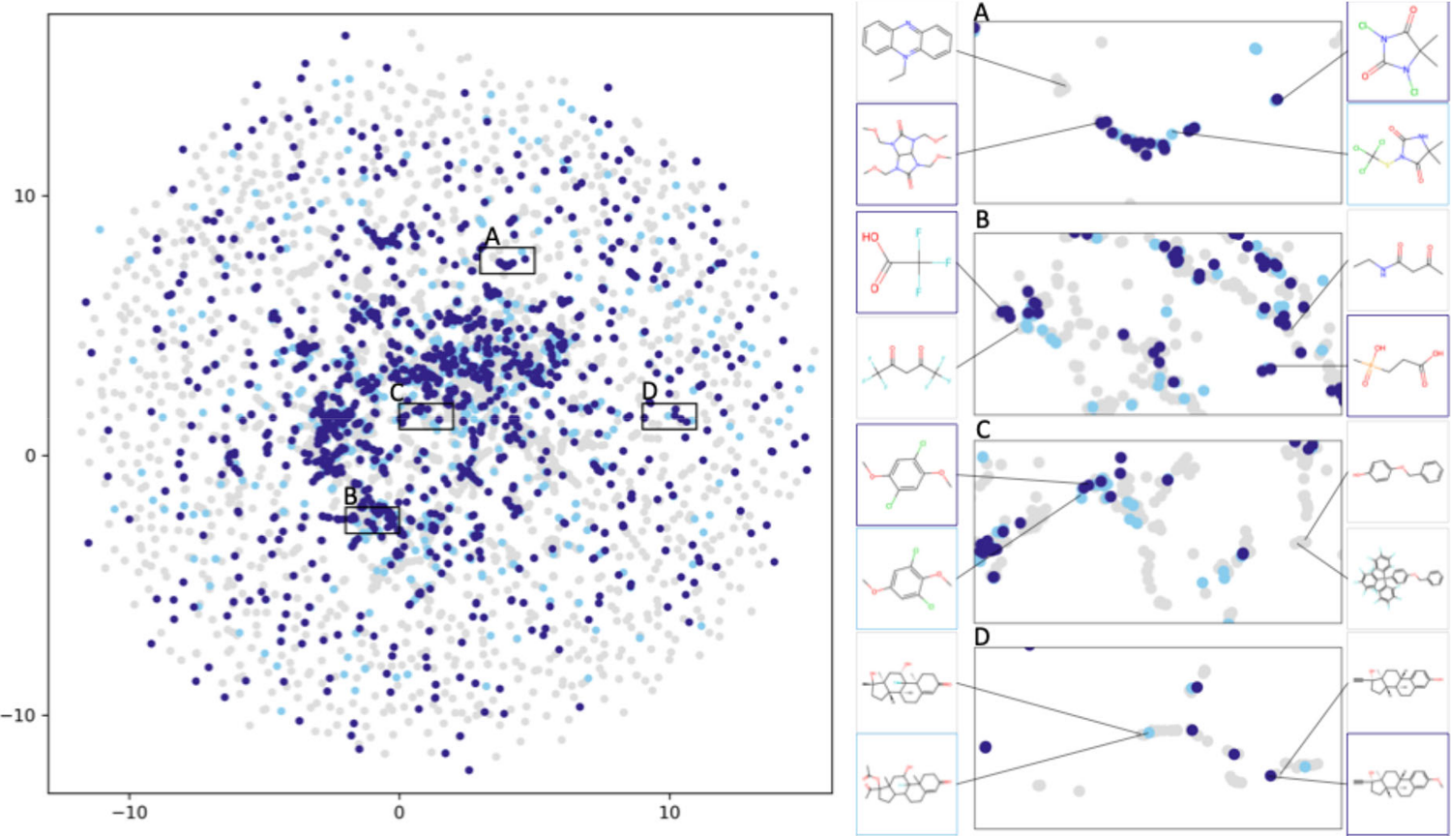
UMAP scatterplot showing the diversity and clustering of molecules in the data set. Pesticide substances with genotoxicity data are highlighted in dark blue, pesticides without genotoxicity in light blue, and nonpesticide substances with genotoxicity data in gray.

Figure 7 highlights the top-10 Murcko scaffolds of pesticide substances that are absent from any genotoxicity data set. These missing Murcko scaffolds occur within the pesticide data set at remarkably low frequencies with occurrence percentages largely below 0.5%, representing only a small fraction of the overall chemical space of pesticide substances. From a regulatory perspective, this suggests that a majority of commonly used or structurally representative pesticide Murcko scaffolds have publicly available genotoxicity test data, and the absence of these rare scaffolds may not represent a substantial data gap. The missing scaffolds follow a pattern: many correspond to structural motifs found in specific pesticide subclasses (e.g., milbemycin-like acaricides, certain heteroaromatic fungicides and insecticides, as well as fused-ring herbicides).

**Figure 7:**
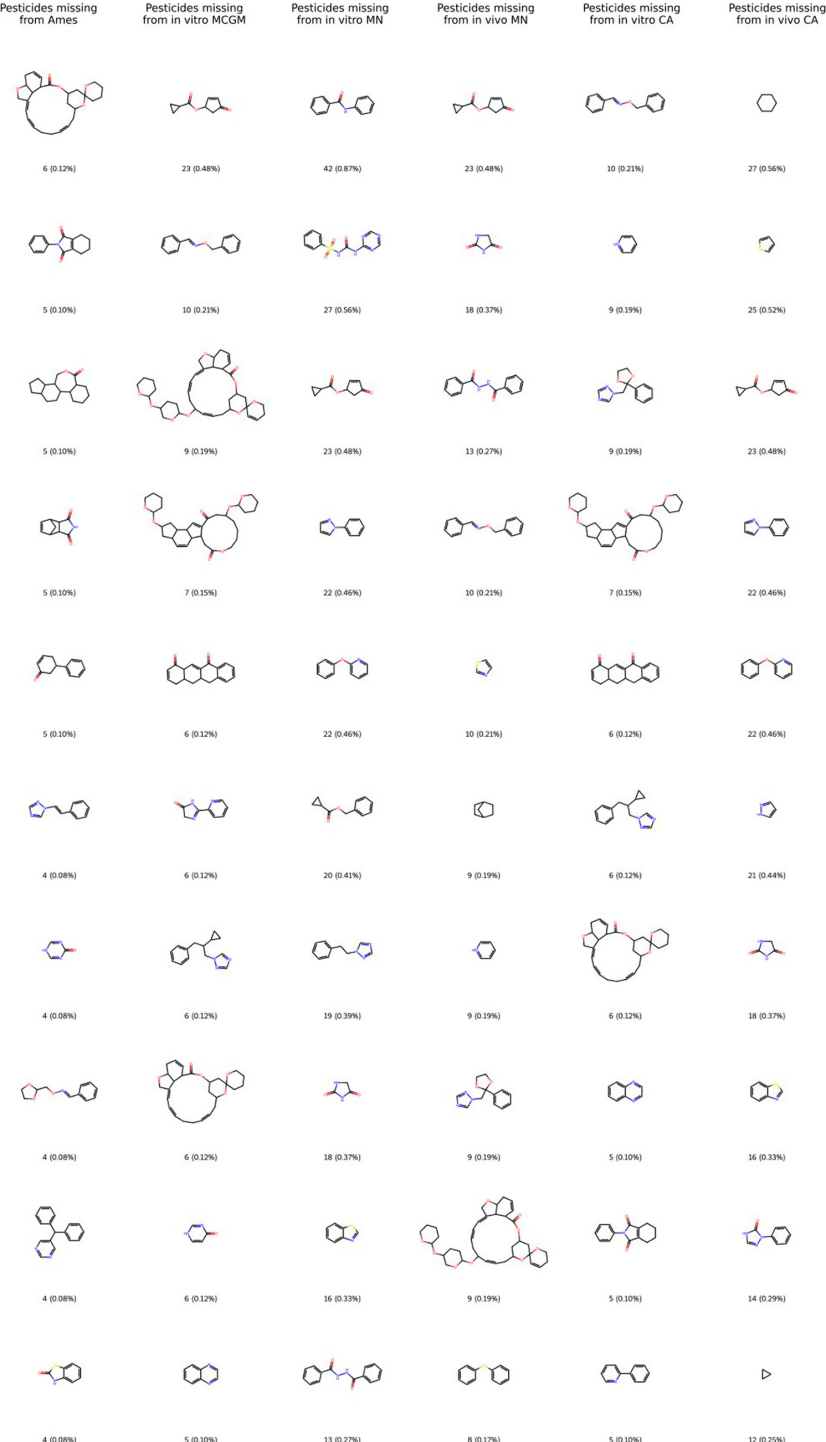
Top-10 most frequent scaffolds present in the pesticide set but not in any of the genotoxicity data sets. The scaffolds are depicted in order of frequency in the pesticide data sets from top to bottom (top = most frequent; bottom = 10th most frequent). Each scaffold is labeled with the count of how many molecules in the pesticide data have that scaffold, and in parentheses with the percentage of the pesticide data set composed of substances with that scaffold.

Structurally, several of the underrepresented motifs feature heteroaromatic or fused-ring systems—motifs that can sometimes be associated with metabolic activation^105,106^ or bioaccumulation potential.^107,108^ These chemotypes are under-represented in the broader pesticide marketplace, which explains their low frequency in the data used here. This lack of data points to “blind spots” in the publicly available databases, representing substances for which there may be insufficient data for hazard determination by nontesting methods. Even low-frequency scaffolds may correspond to specific pesticide substances that can enter the environment or the food chain. Their absence from public genotoxicity data sets highlights the importance of targeted testing and/or data gathering strategies to ensure that rare but structurally distinct chemotypes are not overlooked, particularly when they may exhibit chemical behaviors of concern (e.g., persistence, reactivity). Importantly, this absence reflects only publicly available databases and should not be interpreted as evidence of missing data in regulatory submissions. While scaffold coverage is broadly reassuring, filling these small structural data gaps would strengthen our understanding of the genotoxic potential of pesticide substances. See Table S4 for the entire set of Murcko scaffolds present in each of the endpoint-level data sets.

Figure 8 illustrates the functional groups over- or under-represented in each genotoxicity data set compared with pesticide substances (active ingredients, metabolites, and impurities). The enrichment of annulated ring systems and amines is consistent across all genotoxicity data sets. From a toxicological standpoint, this observation is highly relevant: annulated rings, including polycyclic aromatic hydrocarbons such as benzo­[*a*]­pyrene, are prototypical genotoxicants with well-documented DNA intercalation and metabolic activation pathways. Their consistent presence reinforces that the genotoxicity data sets capture chemical features of compounds with established genotoxic liability. On the other hand, amines are both structurally diverse and mechanistically ambiguous. Depending on the context, amines might undergo bioactivation to reactive intermediates or remain relatively benign. Their enrichment highlights that amines are both computationally challenging to model and important to scrutinize in regulatory safety assessments. This complexity arises from their context-dependent bioactivation (e.g., CYP-mediated formation of reactive intermediates), sensitivity to protonation state, and structure variability that limits QSAR accuracy.^54,109,110^

**Figure 8:**
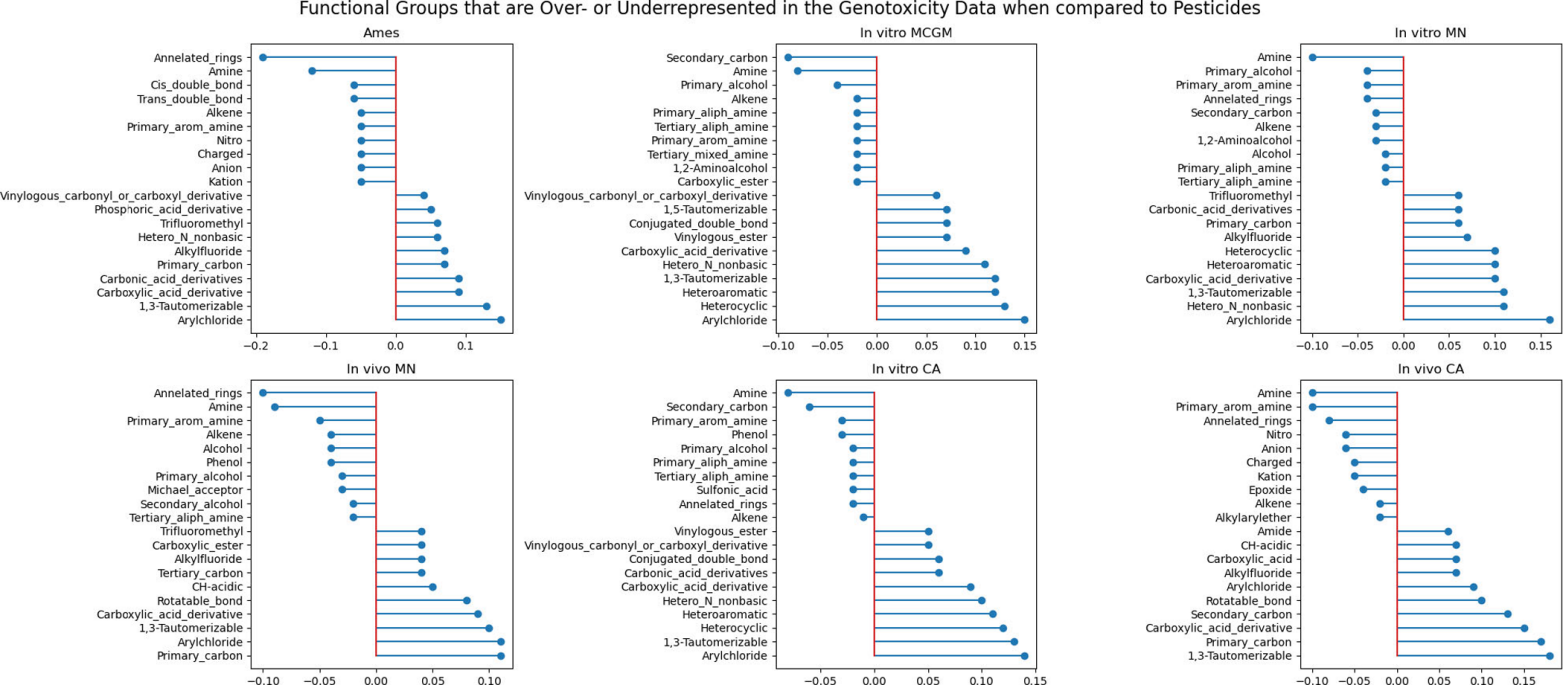
Difference in the proportion of substances containing a functional group in the pesticide set and the proportion of substances with the same functional group in each genotoxicity data set. A number below zero means that the functional group is overrepresented in the genotoxicity data set (as compared to the pesticides). In contrast, a number above zero means that the functional group is underrepresented in the genotoxicity data set (as compared to the pesticides). The top-10 over- and underrepresented functional groups are shown for each data set.

Conversely, functional groups that are systematically underrepresented, including 1,3-tautomerizable motifs (e.g., keto–enol and amide-iminol tautomers), carboxylic acid derivatives, and halogen substituents (e.g., aryl chlorides and alkyl fluorides), reflect structural trends typical of pesticide chemistry. For example, halogenation is widely used in pesticide design to enhance the metabolic stability, but such modifications are less common in benchmark genotoxicants. Carboxylic acid derivatives may confer polarity and rapid clearance, which reduces the likelihood of DNA reactivity. From a regulatory perspective, these differences suggest that genotoxicity databases are enriched with chemotypes that increase the likelihood of eliciting DNA damage. In contrast, pesticide libraries incorporate features selected for stability, bioavailability, and agricultural utility. See Table S5 for a complete list of functional groups present across all endpoint-level data sets.

These imbalances also have important implications for QSAR model development and application. Modeling procedures that rely heavily on data sets enriched in annulated rings and amines may overfit to these structural classes, inflating apparent performance while limiting generalizability. Conversely, underrepresented groups, such as halogenated scaffolds common in pesticides, may fall outside the model’s applicability domain, decreasing the utility of predictions when they are used to assess impurities or metabolites of pesticide candidates. Such biases may reduce confidence in QSAR predictions during regulatory review, particularly if the training data do not reflect the structural space of the compounds under evaluation. Addressing these representational gaps, for instance, by creating QSAR models with targeted training data sets representing specific chemotypes relevant to the query substance (see, for instance, the AMINES data set and QSAR model),^54^ could help build confidence in the use of QSAR predictions in regulatory decision-making.

## Conclusions

In this work, we compiled comprehensive data sets of known pesticides (active substances, metabolites, and impurities) and compared them with substances represented in genotoxicity data sets to characterize the intersection of their chemical space. As a second step, we evaluated how this intersection varies, depending on the genotoxicity test method. Pesticides are, by definition, toxic substances; their toxicity toward their respective target organisms is the very property that makes them valuable to us. Nonetheless, to protect human and environmental health, undesired general or off-target toxicity precludes the use of these substances. Coverage of the pesticide chemical space by reliable data can be considered as a prerequisite for predictive toxicology methods, including QSAR or read-across methods, and should thus be understood to provide the necessary confidence in those methods.

Genotoxicity is a crucial toxicological end point, and evidence of the (lack of) genotoxicity of pesticides and biocides is required for their approval by regulatory authorities. Nonetheless, genotoxicity test data are disclosed to the public in a nonuniform manner. For example, the renewal assessment report for glyphosate contains more than 100 genotoxicity study reports.^111^ For pesticides that attract less attention, finding genotoxicity test data in the public domain may be challenging due to their impurities or metabolites. Therefore, it is desirable to better characterize the overlap between public genotoxicity data sets and those of pesticides, particularly, and to understand how this relationship differs for different genotoxicity test methods. This is important for the accurate and targeted use of databases, including the training and testing of QSAR models, in line with regulatory requirements. In addition, this analysis can guide data generation and gathering activities in less well-covered domains.

In total, there were 4826 pesticide substances from 18 data sets. From 19 genotoxicity data sets, 19,897 substances had at least one test result in one of the six genotoxicity study types evaluated in this study. Data for the Ames test were the most abundant. With 18,954 substances (of which 1974 are pesticide substances), both the physicochemical and fingerprint-based chemical space of pesticides is well covered by the Ames test. The in vitro CA test, with 3841 substances (1045 pesticides), had the second most data among genotoxicity tests, followed by the in vitro MCGM test, with 3312 substances (955 pesticides). Although the data for these two tests covered a large portion of the pesticide chemical space, there were regions on the margins where the test data were absent.

For the two micronucleus tests, intermediate coverage of the pesticide chemical space could be shown. The in vivo MN and in vitro MN tests had 1452 substances (636 pesticides) and 1329 substances (399 pesticides), respectively. The in vivo CA test had data for the fewest substances, with only 319 substances (166 pesticides). For the in vivo CA test, only small, specific regions of the pesticide chemical space were covered by the genotoxicity data. These observations supports earlier discussions of limited (public) data availability as the reason for the limited performance of QSAR models for predicting chromosome damage^112^ and are in line with a previous regulatory decision taken by EFSA and EU member state representatives “that the predictions for end points different than Ames still could be used as part of the weight of evidence approach, but not individually, considering their limited reliability.^104^

Nevertheless, for all the genotoxicity tests, the major pesticide scaffolds were represented within the genotoxicity data, with the most frequent individual pesticide scaffolds that were missing accounting for between 0.9 and 0.1% of pesticide substances each, depending on the genotoxicity test. A distinct pattern could be discerned when the frequency of functional groups between the substances with genotoxicity data and the pesticides was compared. Prototypical genotoxins were frequently over-represented in the genotoxicity data, including annulated rings (e.g., polycyclic aromatic hydrocarbons), amines (especially primary aromatic amines), nitro groups, and charged species. On the other hand, functional groups are frequently underrepresented in the genotoxicity data compared to pesticides that include stereotypical moieties, such as alkyl fluoride and aryl chloride groups. This should be taken into consideration when initiating data generation or gathering activities aimed at improving the regulatory acceptance of predictive methods.

Given the legal requirements for the genotoxicity testing of pesticides, it is somewhat surprising that many pesticide substances lack test data, at the very least for the basic in vitro battery of genotoxicity tests. It is unlikely that these data do not exist. Instead, these data are often disclosed outside of the scientific literature and, hence, are less likely to have been discovered by the creators of the genotoxicity data sets used here. Thus, going forward, industry and regulatory authorities must work together to ensure that these data are consistently and reliably disclosed to promote and enable further research into the genotoxic potential of pesticide substances. One prominent example of such a data-sharing effort is the EFSA Pesticides Genotoxicity Database,^31^ which was recently extended^16^ to include updated genotoxicity data in a broader array of pesticide active substances, impurities, and metabolites. These data-sharing initiatives are crucial for the development of in silico methods for the prediction of genotoxicity. As AI continues to advance, ever more powerful algorithms require ever-increasing amounts of data, with the promise of better predictions of genotoxicity.

Although the analysis presented here provides a comprehensive overview of the chemical space overlap of pesticide and genotoxicity data sets, several further considerations must be taken into account. Our approach primarily focused on physicochemical properties and molecular fingerprints and therefore did not include additional biological dimensions relevant to genotoxicity. Combining chemical space analysis with information about the mode(s) of action of each substance or with metabolic pathway data could provide a more holistic understanding of genotoxic potential and help refine or validate rule-based predictive systems. However, the standard genotoxicity test systems used here do not typically provide direct evidence regarding the mode of action, and additional curated data would be required to perform such analyses. Several new in vitro genotoxicity test systems that provide mechanistic information have recently been developed and/or validated.^113–115^

Two further caveats to the work presented here involve the conservative criteria for making genotoxicity hit calls and the issue of data modification resulting from collecting data from different sources. The use of conservative criteria, whereby any positive result triggers a “positive” classification, reflects a cautious regulatory approach intended to minimize false negatives. However, this may also amplify the perception of genotoxic risk in cases in which positive results are isolated or uncertain. A more refined weight-of-evidence methodology—considering study quality, reproducibility, and mechanistic relevance—would necessitate a detailed examination of the original studies, which were largely unavailable in the present analysis. Moreover, the integration of data from multiple sources carries an inherent risk of unintentional data modification, such as transformations of SMILES representations, desalting procedures, and discrepancies in chemical structure depiction. Such modifications can substantially artificially inflate the data set size without the addition of new experimental results. In one recent example, the data set size increased by about 30% due to the aforementioned modifications.^116^ While every effort was made to minimize such effects in this work, this potential source of variability warrants further investigation and transparent reporting in future studies.

In conclusion, chemical space analysis has great potential to contribute to regulatory toxicology, in general, beyond genotoxicity. As the field seeks to move away from animal testing and toward novel test methods, validating these new methods is of great importance. Comparing the chemical space of a specific regulatory domain to the set of reference substances used to validate a new model could help ensure that these new test methods are broadly applicable, reveal where results from new methods diverge from traditional methods, and avoid validation with an inappropriate set of reference substances that do not represent the regulated substance sufficiently. Chemical space visualization could aid in decision-making about where it is appropriate or inappropriate to perform a read-across from the active substance or for selecting which substance with data is most well-suited to be used as the source. Additionally, chemical space represents a powerful tool for understanding where a query substance lies in the training set of a QSAR model, helping to visualize and understand the model’s applicability domain, supporting the confidence-building ultimately needed for broad regulatory acceptance of new methodology.

## Supplementary Material







## Data Availability

All processed data used in this work are available here: 10.5281/zenodo.15463232.

## Notes

Figure S1: UMAP parameters grid search – Trustworthiness vs Butina; Table S1: Substance and scaffold summary information for the DrugBank data; Table S2: Discarded SMILES strings containing wildcard*; Table S3: Genotoxicity data set overlap with pesticide substances and DrugBank by Tanimoto similarity (PDF)
Table S4: Count and percent of substances with each Murcko scaffold in the end point data sets (TXT)
Table S5: Fraction of substances with each functional group in the processed end point data sets (TXT)
