# Supplementary material for: Comparative Chemical Space Analysis of Pesticides and Substances with Genotoxicity Data
Source: Chem Res Toxicol. 2025 Oct 29;38(11):1871–88. doi: 10.1021/acs.chemrestox.5c00198 (PMC12628336; doi:10.1021/acs.chemrestox.5c00198)
Supplement: Supplementary file 1 [file tx5c00198_si_001.pdf]

Supporting Information

# Comparative Chemical Space Analysis of Pesticides and Substances with Genotoxicity Data

Daniel H. Foil,[1,2] Jeannette König,[1] Kristin Herrmann,[1] Roxane A. Jacob,[2,3] Carsten Kneuer,[1*] Johannes Kirchmair[3*]

[1] Department of Pesticides Safety, German Federal Institute for Risk Assessment, Max-Dohrn-Str. 8-10, 10589 Berlin, Germany
[2] Vienna Doctoral School of Pharmaceutical, Nutritional and Sport Sciences, University of Vienna, Josef-Holaubek-Platz 2, 1090 Vienna, Austria
[3] Department of Pharmaceutical Sciences, Division of Pharmaceutical Chemistry, Faculty of Life Sciences, University of Vienna, Josef-Holaubek-Platz 2, 1090 Vienna, Austria

* Corresponding authors: carsten.kneuer@bfr.bund.de, johannes.kirchmair@univie.ac.at

# Supporting Information Table of Contents


| | Page |
|---|---|
| Figure S1: Trustworthiness and sensitivity analysis for Butina vs UMAP grid search | S3 |
| Table S1: Substance and Scaffold summary information for the DrugBank data | S3 |
| Table S2: Discarded SMILES strings containing wildcard * | S4 |
| Table S3: Genotoxicity data set overlap with pesticide substances and DrugBank by Tanimoto similarity | S5 |


**Further supporting information is available in CSV file format:**

Table S4: Count and percent of substances with each Murcko scaffold in the endpoint data sets

Table S5: Fraction of substances with each functional group in the processed endpoint data sets

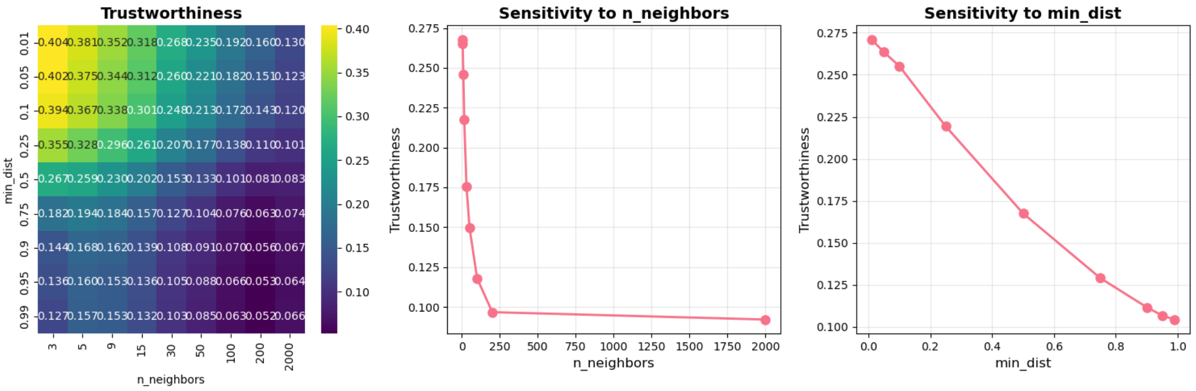


Figure S1: Trustworthiness and sensitivity analysis for Butina vs UMAP grid search

Table S1: Substance and Scaffold summary information for the DrugBank data

| Data set | Substances (initial) | Substances (curated) | Murcko Scaffolds | Mean molecules per Murcko (SD) | Murcko singletons (singletons per scaffold) |
|---|---|---|---|---|---|
| DrugBank Approved Drugs | 2,647 | 2,610 | 1,338 | 1.65 (6.53) | 1,089 (0.81) |
| DrugBank (All) | 12,299 | 12,155 | 6,013 | 1.78 (12.40) | 4,933 (0.82) |

Table S2: Discarded SMILES strings containing wildcard *

| SMILES | Source data set | DSTXSID |
|---|---|---|
| OCCN(CCO)CCO.CCCCCCCCCCCC*.OS(=O)(=O)C1=CC=CC=C1 | CP_DAT | DTXSID5027932 |
| CCCCCCCCCC*.OS(=O)(=O)C1=CC=CC=C1 | PESTHHBS | DTXSID2028723 |
| *-CC(-*)N1CCCC1=O | EPAPCS | DTXSID0025941 |
| O*.OS(=O)(=O)C1=CC=CC=C1 | EPAPCS | DTXSID80895032 |
| CCCCCCCCCC*.OS(=O)(=O)C1=CC=CC=C1 | EPAPCS | DTXSID2028723 |
| C*.C*.OS(=O)(=O)C1=CC=CC=C1 | EPAPCS | DTXSID3026302 |
| OCCN(CCO)CCO.CCCCCCCCCCCC*.OS(=O)(=O)C1=CC=CC=C1 | EPAPCS | DTXSID5027932 |
| [Na+].CCCCCCCCCCC(C)*.[O-]S(=O)(=O)C1=CC=CC=C1 | EPAPCS | DTXSID50895044 |
| [Na+].CCCCCCCCC*.[O-]S(=O)(=O)C1=CC=CC=C1 | EPAPCS | DTXSID60895050 |
| [Cl-].[Cl-].C[N+](C)(CC-*)CC[N+](C)(C)CCO-* | EPAPCS | DTXSID8034829 |
| [Cl-].C[N+]1(C)CC(C-*)C(C-*)C1 | EPAPCS | DTXSID8035714 |
| [Na+].CCCCCCCC*.[O-]S(=O)(=O)C1=CC=CC=C1 | EPAPCS | DTXSID80895032 |
| O*.OS(=O)(=O)C1=CC=CC=C1 | EPAOPPIN | DTXSID1027390 |
| CCCCCCCCCC*.OS(=O)(=O)C1=CC=CC=C1 | EPAOPPIN | DTXSID2028723 |
| C*.C*.OS(=O)(=O)C1=CC=CC=C1 | EPAOPPIN | DTXSID3026302 |
| *-CCCCCCCCCCCC(=O)N-* | EPAOPPIN | DTXSID3040803 |
| OCCN(CCO)CCO.CCCCCCCCCCCC*.OS(=O)(=O)C1=CC=CC=C1 | EPAOPPIN | DTXSID5027932 |
| [Na+].CCCCCCCCCCC(C)*.[O-]S(=O)(=O)C1=CC=CC=C1 | EPAOPPIN | DTXSID50895044 |
| [Na+].CCCCCCCCC*.[O-]S(=O)(=O)C1=CC=CC=C1 | EPAOPPIN | DTXSID60895050 |
| [Cl-].[Cl-].C[N+](C)(CC-*)CC[N+](C)(C)CCO-* | EPAOPPIN | DTXSID8034829 |
| [Cl-].C[N+]1(C)CC(C-*)C(C-*)C1 | EPAOPPIN | DTXSID8035714 |
| *-CC(-*)N1CCCC1=O | EPAOPPIN | DTXSID0025941 |
| [Na+].CCCCCCCC*.[O-]S(=O)(=O)C1=CC=CC=C1 | EPAOPPIN | DTXSID80895032 |

Table S3: Genotoxicity data set overlap with pesticide substances and DrugBank by Tanimoto similarity

| **Data set** | **Overlap with pesticide substances similarity > 0.75** | **Overlap with approved drugs of DrugBank similarity > 0.75** | **Overlap with DrugBank (all) substances similarity > 0.75** |
|---|---|---|---|
| AMINES | 27 (4%) | 27 (4%) | 53 (9%) |
| AmesFormer | 1,088 (11%) | 833 (9%) | 1,468 (15%) |
| Benfenati | 683 (12%) | 410 (7%) | 810 (14%) |
| BfR | 422 (100%) | 5 (1%) | 30 (7%) |
| CAESAR | 608 (14%) | 392 (9%) | 767 (18%) |
| CORAL | 181 (38%) | 100 (21%) | 165 (35%) |
| ECVAMneg | 110 (55%) | 62 (31%) | 97 (49%) |
| ECVAMpos | 145 (22%) | 83 (13%) | 131 (20%) |
| EFSA_PestGentox | 697 (100%) | 20 (3%) | 60 (9%) |
| Hansen | 808 (12%) | 535 (8%) | 1,001 (15%) |
| IRFMN | 586 (48%) | 213 (18%) | 358 (30%) |
| ISS | 250 (38%) | 138 (21%) | 200 (30%) |
| ISSMIC | 93 (34%) | 53 (20%) | 87 (32%) |
| ISSSTY | 208 (13%) | 76 (5%) | 168 (11%) |
| Karamertzanis | 1,868 (15%) | 705 (6%) | 1,550 (13%) |
| OCHEM | 1,226 (13%) | 1,242 (13%) | 1,959 (20%) |
| SARPY | 608 (14%) | 392 (9%) | 767 (18%) |
| VERMEER | 103 (27%) | 76 (20%) | 119 (32%) |
| Xu | 916 (12%) | 620 (8%) | 1,145 (16%) |
| Total | 2,793 (14%) | 1,732 (9%) | 3,052 (15%) |
